# Telesurgery of Microscopic Micromanipulator System “NeuRobot” in Neurosurgery: Interhospital Preliminary Study

**DOI:** 10.4137/jcnsd.s2552

**Published:** 2009-07-17

**Authors:** Tetsuya Goto, Takahiro Miyahara, Kazutaka Toyoda, Jun Okamoto, Yukinari Kakizawa, Jun-ichi Koyama, Masakatsu G. Fujie, Kazuhiro Hongo

**Affiliations:** 1 Department of Neurosurgery, Shinshu University School of Medicine, Matsumoto, Japan.; 2 Faculty of Science and Engineering, Waseda University, Tokyo, Japan.; 3 Graduate school of Science and Engineering, Waseda University, Tokyo, Japan. Email: khongo@shinshu-u.ac.jp.

**Keywords:** neurosurgery, robotic surgery, telesurgery, telemedicine, manipulator

## Abstract

**Object:**

Robotic surgery can be applied as a novel technology. Our master-slave microscopic-micromanipulator system (NeuRobot), which has a rigid endoscope and three robot-arms, has been developed to perform neurosurgical procedures, and employed successfully in some clinical cases. Although the master and slave parts of NeuRobot are directly connected by wire, it is possible to separate each part and to apply it to telesurgery with some modifications. To evaluate feasibility of NeuRobot in telesurgery, some basic experiments were performed.

**Methods:**

The quality of telemedicine network system between Shinshu University and one of the affiliated hospitals, which was completely separated from other public network systems, was investigated. The communication delay was calculated from the transmitting and the receiving records in the computers set in each hospital. The relationship between the change in communication delay from the master part to the slave part of NeuRobot (0, 100, 300, 500 and 700 ms) respectively and feasibility of NeuRobot was investigated. The task performance time in each time changing group was compared. Feasibility of NeuRobot in telesurgical usage was evaluated. The master part and the slave part of NeuRobot placed in each hospital were connected through private network system. Interhospitally connected NeuRobot was compared with directly connected one in terms of task performance time.

**Results:**

Less than 1 ms was required for corresponding the data in a steady transmitting state. Within 2 seconds after connection, relative time delay (maximum 40 ms) and packet loss were sometimes observed. The mean task performance time was significantly longer in over 500 ms delayed group compared with directly connected NeuRobot. There was no significant difference in the task performance time between directly connected NeuRobot and interhospitally connected NeuRobot.

**Conclusion:**

Our results proved that telesurgical usage of NeuRobot was feasible. Telesurgical usage of telecontrolled manipulator system is recommended for application in a private network system in order to reduce technical and ethical problems. Some technical innovations will bring breakthrough to the telemedicine field.

## Introduction

Application of robotic technology in surgery is a recent innovation. Da Vinci^®^ (Intuitive Surgical, Inc., Sunnyvale, CA) and Zeus^®^ (Computer Motion, Inc., Goleta, CA), which are robotics telecontorolled manipulation system performing several surgical tasks, have been used widely in the clinical field.^1^,^10^ In microneurosurgical field, NeuRobot, telecontrolled microscopic-micromanipulator system, has been developed to perform neurosurgical procedures, and has been applied successfully in some clinical cases.^2^–^6^,^9^

Telemedicine, definitive as a medical behavior apart from each other, has become a necessity for medical treatment in distant locations. Telecontrolled manipulation system has a possibility for telemedicine, in which patients are surgically treated by a surgeon situated distantly.^7^,^8^,^11^ If telesurgical usage of the telecontrolled manipulation system is feasible, it is extremely beneficial not only for the patient but also for the surgeon. Although da Vinci already achieved the telesurgical usage on clinical level,^8^ application of the telecontrolled manipulator system to telesurgery encounters some difficulties. In telesurgery, a patient and a surgeon are not in the same place, and surgical procedures must be conducted through the network system. In this situation, communication delay from controlling the manipulator to confirming the movement of the manipulator must exist.^11^ The communication delay may affect the feasibility and safety of manipulation. In this paper, in order to evaluate the possibility of the telesurgical usage of NeuRobot, several basic examinations were performed.

## Materials and Methods

**Figure 1:**
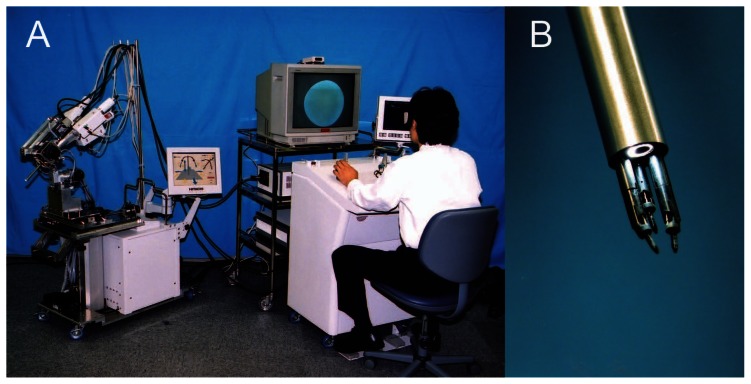
**A**) Photograph of the whole view of the NeuRobot: micromanipulator and manipulator-supporting device (left), display monitor (center), and operation input device (right). **B**) The tip of the insertion cylinder: micro-forceps at the bilateral robot-arms and the potassium titanyl phosphate laser fiber at the center robot-arm.

The characteristic of NeuRobot is to perform a surgical operation thorough a small burr hole. The NeuRobot was composed of four parts, 1) micromanipulator, 2) manipulator-supporting device, 3) operation input device, and 4) display monitor (Fig. 1). The main feature of NeuRobot was a 10-mm diameter and 17-cm length of rigid insertion cylinder containing a three dimensional (3D) endoscope, three robot-arms, and five irrigation and suction channels. This system was designed so that the surgeon can remotely operate the slave manipulator by controlling levers on the operation-input device, while watching a 3D monitor. Each robot-arm had three degrees of freedom (rotation, neck swinging, and forward/backward motion). All movements of the micromanipulator were controlled from the operation input device with an accuracy of a 0.02-mm order and no instability. Micromanipulator and operating input device are directly connected by wire, and they controlled each other by signals exchange in 30 cycles per second from many angle sensors in joints of the micromanipulator and the handles of the operation input device. Not only the signals from the handle control the micromanipulator, but also the signals from joints of the micromanipulator control the movement of the handles. The surgeon could feel the precise movements of the micromanipulator from the position of the handles. The NeuRobot could be accurately controlled by a remotely located surgeon with adequate degrees of freedom; basic procedures, such as dissecting, cutting, coagulating, stitching, and tying procedures, could be performed. The target of pathology is a tumor with a volume of 1 cm^3^. The NeuRobot was capable of performing finely controlled motions which the human hand cannot and, thus, provided more delicate surgical procedures.^2^–^6^,^9^

## Modification of NeuRobot for Telesurgery

**Figure 2:**
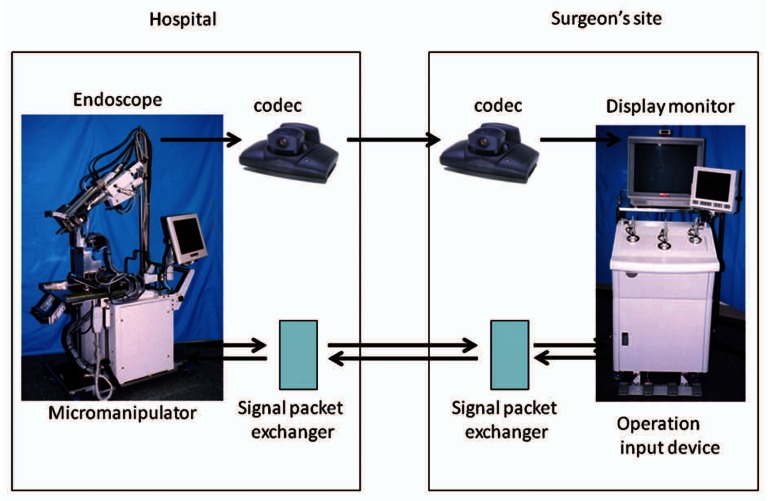
Illustration of NeuRobot for telesurgery: Left square showing a hospital and right square showing surgeon’s operation site. The 3D endoscopic images converted by codec is transmitted and reaches to a surgeon’s sitel. Micromanipulator in a hospital is controlled by the operating input device in surgeon’s site. The signal between the micromanipulator and operation input device changed to the packet by the signal packet exchanger is transmitted to each hospital.

For telesurgical usage, the signal between the micromanipulator and operation input device must be adjusted to the packet to correspond within the network system. The signal packet exchanger had been newly adopted for NeuRobot. The signal packet exchanger changed the signal to 400 bytes of User Data Protocol (UDP) packet and transmited in 20 cycles per second, which gave 64 kilobit per second (kbps) connection rate. NeuRobot was re-programmed to stop the movements of micromanipulator automatically for safety reason when the manipulator did not receive a consecutive 5 packets (250 ms) between the micromanipulator and operation input device (Fig. 2).

The 3D endoscopic image must also be transmitted from the operative field to the surgeon. A codec, a device capable of encoding and decoding a digital data stream, was necessary for image transmission in the network system. Codec converted the continuous 3D endoscopic image from analog signal to digital data. It was encoded, transmitted, decoded, and was re-converted to analog signal. ViewStation FX (Polycom Inc. CA, USA) was used as a telepresence system for NeuRobot (Fig. 2). ViewStation FX had abilities as follows: bandwidth was 1920 kbps, Images were transmitted by H.264 system and UDP transmission, The required time to convert an image was 200 ms (unpublished data, accuracy: 10 ms), and image resolution decreased when the signal was over the bandwidth, but converting time was maintained stable.

## Interhospital Network System

The private network system for telemedicine has been established between Shinshu University Hospital and several affiliated hospitals, public institutes in Nagano Prefecture. Each institute was connected by 155 megabites optical fiber lines and 100 BASE-TX switching hubs. The network system was exclusively used for telemedicine and was completely separated from other public network systems. Ohmachi Municipal Hospital, which was one of the affiliated hospitals and was 40 km away from Shinshu University Hospital, was selected in this examination.

To evaluate the feasibility of these systems, three subclinical examinations were checked; 1: Evaluation of interhospital network system, 2: Effect of time delay, 3: Actual interhospital usage of NeuRobot.

## Results

### Method: Examination 1: Evaluation of interhospital network system

The quality of private telemedicine network system between Shinshu University and Ohmachi Municipal Hospital was evaluated. Two computer systems, which were set in each hospital, were connected to the telemedicine line. The time in each computer system was synchronized with 0.05 ms accuracy by utilizing the global positioning system.^7^ A total of 70,000 packets were transmitted in 20 cycles per second. The packet size was 400 and 500 bytes. This examination was repeated 12 times. The communication delay and packet loss were investigated by the required time for transmitting and receiving of the packet recorded in each computer system.

### Result: Examination 1

0.8 ms was required for corresponding the 400 bytes UDP packet and 0.9 ms was needed for corresponding the 500 bytes UDP packet in a steady data communication state. In the initial connection phase (maximum 2 seconds after connection), 3 times of relative time delay and one time of packet loss were observed in 12 times of connection. 3 times of maximum time delay were 4 ms, 5 ms and 60 ms, respectively. One time of packet loss (consecutive 6 packets) was observed in 60 ms time delay of the initial connection phase.

### Methods: Examination 2: Effect of the time delay

**Figure 3:**
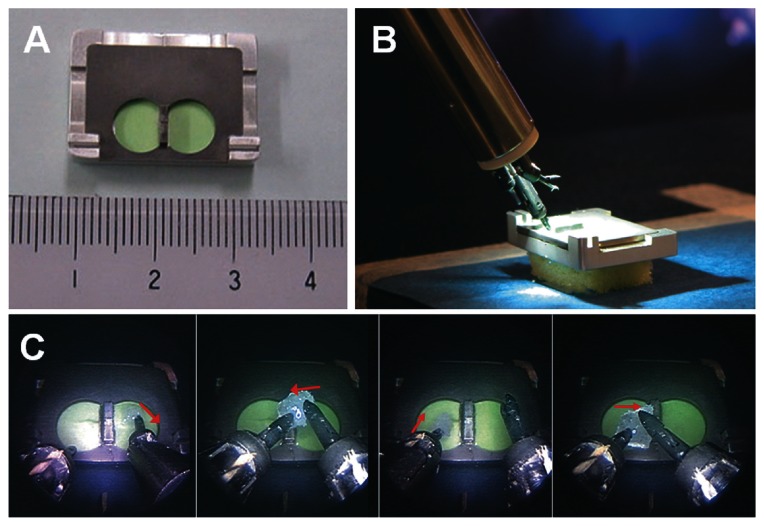
Photographs of the experiment 2 and 3: **A**) The task field and the scale in millimeters. **B**) A whole view of the task field and micromanipulator during the task procedure. **C**) Four procedures in one task session from the endoscopic view: (1) Pinching up the cotton by one robot-arm from the hollow, (2) Taking a piece of cotton to another robot-arm, (3) Placing the cotton to another hollow, (4) Returning the cotton to the previous hollow by the procedures 1 to 3.

Repeatable task was prepared for investigation of relationship between usability of NeuRobot and communication delay from controlling the handle to confirming the movement of the micromanipulator. The task field was composed of two parts: 7-mm diameter and 1-mm depth hollows, and 2-mm height of septum wall between the hollows. The task in one session consisted of 4 procedures: 1) pinching up the 1 mm^3^ cubic cotton piece by one robot-arm from the hollow, 2) taking the cotton to another robot-arm, 3) placing it to another hollow, 4) returning the cotton to the previous hollow by repeating procedures 1 to 3 (Fig. 3).

Four neurosurgeons, who were skilled in operating the NeuRobot, but were not informed regarding the task, joined the examination. Operation input device and micromanipulator were set in the laboratory room. Firstly, each neurosurgeon continuously performed 5 sessions of the task on the directly connected NeuRobot (0 ms time delay). Secondly, the communication time from the operation control device to micromanipulator was intentionally delayed (100 ms, 300, 500, and 700 ms). Each 4 neurosurgeon continuously performed 3 sessions of the task on the same time delayed NeuRobot.

All procedures were recorded on a digital videotape. The time required, technical quality and subjective difficulty were evaluated. In each time delay group, evaluation by 0–10 scale (0: worst—10: best) about quality of image, impact of time delay on performance, and overall safety of the procedure, were asked to each neurosurgeon. The data were analysed by paired and non paired t-tests.

### Results: Examination 2

All the procedures were successfully performed by the micromanipulator without any manual assistance. Task failures such as cotton dislodgement did not occur. Movement of NeuRobot was accurate and no system error or system down against operator control happened during the examination.

**Figure 4:**
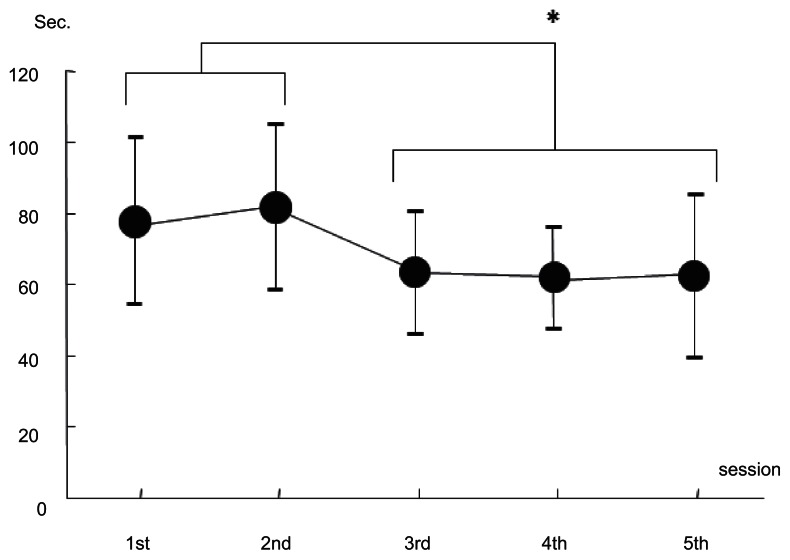
A graph showing means of the task performance time with directly connected NeuRobot in each session and the error bar indicating the standard deviation of the means (n = 4). Asterisk: p < 0.05.

The mean time to perform the task by directly connected NeuRobot was 78 seconds in the first session, 82 in the second, 63.5 in the third, 62 in the fourth, and 62.5 seconds in the fifth session, respectively (n = 4). The mean time in the first and second session was significantly longer than that in the third session or later (Fig. 4).

**Figure 5:**
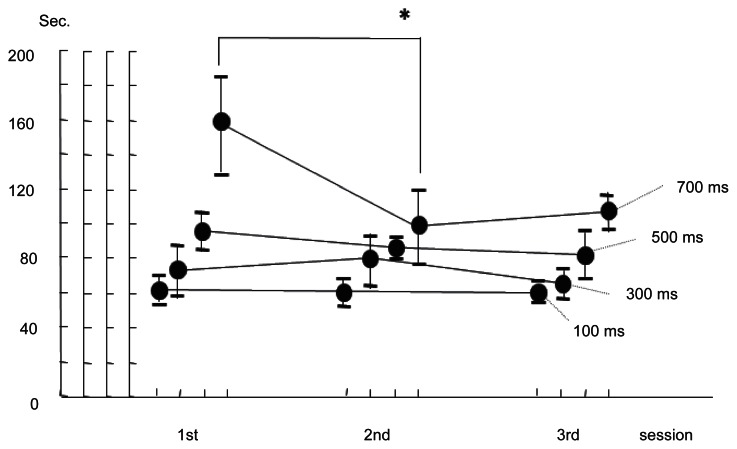
A graph showing means of the task performance time with artificial time delay of 100 ms, 300 ms, 500 ms, and 700 ms in each session and the error bar indicating the standard deviation of the means (n = 4). Asterisk: p < 0.05.

The mean time to perform the task by 100 ms delayed NeuRobot was 62 seconds in the first session, 61 in the second, and 60 seconds in the third session, respectively. The mean time with 300 ms delayed NeuRobot was 73 seconds in the first session, 80 in the second, and 66 seconds in the third session. The mean time with 500 ms delayed NeuRobot was 96 seconds in the first, 87 in the second, and 82 seconds in the third session. The mean time with 700 ms delayed NeuRobot was 157 seconds in the first, 99 in the second, and 107 seconds in the third, respectively (Fig. 5). The mean time in the initial period was significantly longer in 700 ms delay group. It was suggested that learning period was necessary when the time delay was 700 ms.

**Figure 6:**
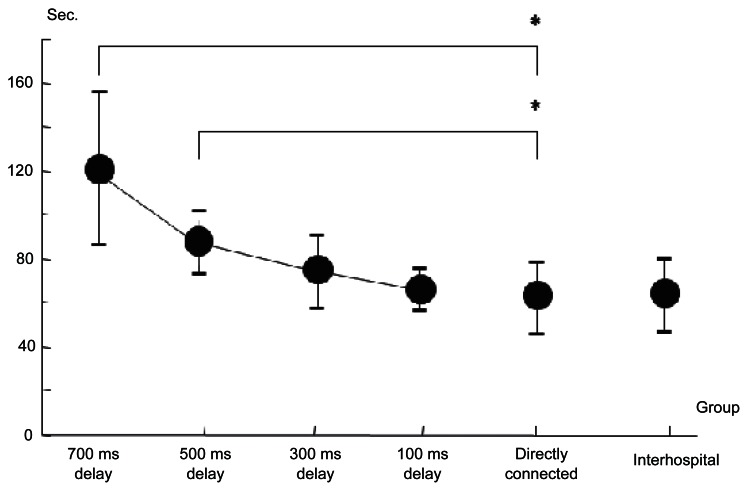
A graph showing means of the task performance time compared with no time delay group and the error bar indicating the standard deviation of the means (n = 12). Asterisk: p < 0.05.

**Figure 7:**
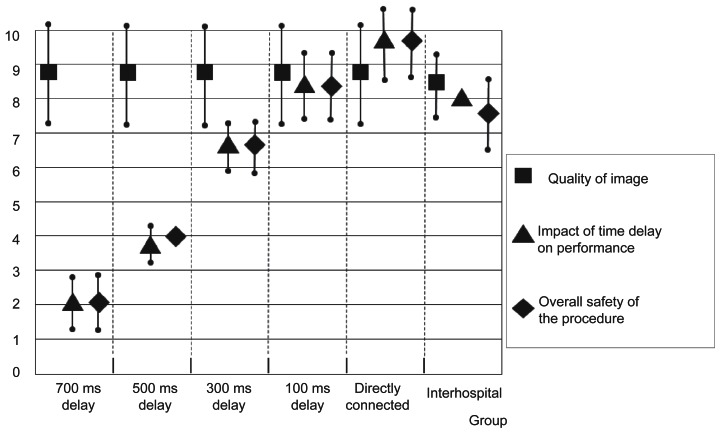
A graph showing means of evaluation by 0–10 scale (0: worst – 10: best) and the error bar indicating the standard deviation of the means (n = 4). About quality of image, impact of time delay on performance, and overall safety of the procedure, which were asked to each neurosurgeon.

Initial two sessions in directly connected group were considered as learning periods. We omitted initial two sessions in directly connected group and the remaining 3 sessions as learned directly connected group were compared with the delay group (n = 12). The significant delay was confirmed in 500 ms and 700 ms delay group (Fig. 6). Figure 7 showed the result of questionnaire. Scale of quality of image was similar in each time delay group. On the contrary, scale of the impact of time delay on performance and overall safety of the procedure decreased with longer time delay. It was considered that using NeuRobot safely beyond 500 ms of time delay was difficult.

### Method: Examination 3: Interhospital usage of NeuRobot

After examination 2, the operating control device was transported to Ohmachi Municipal Hospital and was connected to the manipulator set in the laboratory in Shinshu University through the telemedicine line. Four neurosurgeons same as in the examination 2 operated the micromanipulator in Shinshu University by performing the same task of examination 2 in 3 sessions. Investigation and data analysis were carried out same as in the examination 2.

### Result: Examination 3

All the procedures were successfully performed by the micromanipulator without any manual assistance. No task failures and no system error occurred. The mean time to perform the task was 68 seconds in the first session, 59 in the second, and 58 seconds in the third session, respectively. There was no significant different mean time in performing the task between the learned directly connected group and the interhospital usage group (Fig. 6). The evaluation scale of interhospital group was slightly lower than that of directly connected group; however, it was not significant (Fig. 7).

## Discussion

Telesurgical usage of the telecontrolled manipulation system is a dream.^7^,^8^,^11^ A patient can undertake a surgery at the nearby hospital without going far to a specialized hospital. Even in some clinics at the remote corner of the country, such as isolated island or mountain village, complex surgical procedures may be possible. A surgeon can operate on a patient by manipulating the system from his office even without going to the operation room. Da Vinci^®^ surgical system has been applied to the clinical laparoscopic telesurgery in 2001.^8^ Although the operation was successfully completed, no reports describing the clinical application of telesurgery by telecontrolled manipulator system have been published since ever. Telesurgery by telecontrolled manipulator system has a different problem compared with other types of telemedicine. Whenever the telecontrolled manipulator system is used in telesurgery, two types of data should be transmitted: one is the information of a patient image to a surgeon, and another is the surgeon’s system controlling data to a manipulator in a patient’s hospital. Even if the transmitted data in the telesurgery system is not severely deteriorated composed with those in the directly connected data, it must directly affect feasibility of the manipulator, and thus affects the results of surgery.

Time delay between controlling the system and confirming the movement of the system according to data transmitting is a key issue in telesurgery. Although less than 300 ms of time delay in telerobotic surgery is generally accepted, permissible range of time delay is different in each robotic system. Because surgical manipulations performed by robot are different in each system. We confirmed that time delay was directly correlated with task performance time. We concluded that the NeuRobot could be used with the time delay of less than 500 ms. Communication delay in our network system was less than 1 ms. On the other hand, the codec converting time of 3D endoscopic video image wasted 200 ms. This result means that the ability of the system in telesurgery is influenced by the ability of the codec. To use faster codec is most important to minimize the time delay. The evolution of the telecommunication field will resolve this problem.

Instability during transmission of the packet is also a problem in telesurgery. It is known as “jitter” and it increases when “traffic” in the network system is crowded. The “jitter” means occasional packet loss and wavering of packet sending time and is frequently observed in the public net systems.^7^ Occasional packet loss may cause interference of telesurgery. NeuRobot is safe against packet loss, because the movement of NeuRobot stops automatically and mis-movement did not occur. On the other hand, wavering of packet sending time may be troublesome. The effect of wavering time delay against operation of manipulator is similar to turning a steering wheel during driving a car. We can drive safely as we recognize the relationship between the degree of the turning steering wheel and the changing the direction. If the relationship is frequently changed, nobody can drive safely. To prevent “jitter”, “traffic” in the network must be controlled. In our private network system, no wavering of packet sending time was confirmed in examination 1. It may mean telesurgery should be conducted only in the private network system.

The economical and ethical aspects of telesurgery must be considered before routine clinical usage. Responsibility and liability to a patient are vague in telemedicine. Introducing the telecontrolled robotic systems for each specialized surgery to a hospital has cost expensiveness.^1^ Lack of the presence and an illusion, which an operator may have that he does not operate on a patient but plays a video game, might have some problems. The security in the public net systems is not yet warranted. Many secure barriers like “firewall” may make further time waste during data transmission. Of course, the problem of security can be dissolved by using the private network system.

The establishment of standardized task outcome measures can allow for comparison between different robotic systems. A discussion on this subject should be pertinent as it will allow for more objective and consistent comparisons. There are no standardized tasks in micro-neurosurgical field available in the literature. In this paper, we have arranged to describe standardized task for NeuRobot. We hope that our tasks can be used as micro-neurosurgical task for the comparison between other systems.

## Conclusion

Interhospital usage of NeuRobot, in which telesurgery system has been newly developed, was evaluated in the private network system. Although the best specification of the system has not been established yet, there were no significance differences in maneuverability and safety compared to direct wire connection of NeuRobot. Telesurgical usage of telecontrolled manipulator system is recommended to use in the confident network system in order to reduce many technical and ethical problems. Further technical innovations will bring breakthrough to the telemedicine field.
